# ComprehENotes, an Instrument to Assess Patient Reading Comprehension of Electronic Health Record Notes: Development and Validation

**DOI:** 10.2196/jmir.9380

**Published:** 2018-04-25

**Authors:** John P Lalor, Hao Wu, Li Chen, Kathleen M Mazor, Hong Yu

**Affiliations:** ^1^ College of Information and Computer Sciences University of Massachusetts Amherst, MA United States; ^2^ Psychology Department Boston College Chestnut Hill, MA United States; ^3^ Meyers Primary Care Institute University of Massachusetts Medical School / Reliant Medical Group / Fallon Health Worcester, MA United States; ^4^ Department of Computer Science University of Massachusetts Lowell, MA United States; ^5^ Department of Medicine University of Massachusetts Medical School Worcester, MA United States; ^6^ Bedford Veterans Affairs Medical Center Center for Healthcare Organization and Implementation Research Bedford, MA United States

**Keywords:** electronic health records, health literacy, psychometrics, crowdsourcing

## Abstract

**Background:**

Patient portals are widely adopted in the United States and allow millions of patients access to their electronic health records (EHRs), including their EHR clinical notes. A patient’s ability to understand the information in the EHR is dependent on their overall health literacy. Although many tests of health literacy exist, none specifically focuses on EHR note comprehension.

**Objective:**

The aim of this paper was to develop an instrument to assess patients’ EHR note comprehension.

**Methods:**

We identified 6 common diseases or conditions (heart failure, diabetes, cancer, hypertension, chronic obstructive pulmonary disease, and liver failure) and selected 5 representative EHR notes for each disease or condition. One note that did not contain natural language text was removed. Questions were generated from these notes using Sentence Verification Technique and were analyzed using item response theory (IRT) to identify a set of questions that represent a good test of ability for EHR note comprehension.

**Results:**

Using Sentence Verification Technique, 154 questions were generated from the 29 EHR notes initially obtained. Of these, 83 were manually selected for inclusion in the Amazon Mechanical Turk crowdsourcing tasks and 55 were ultimately retained following IRT analysis. A follow-up validation with a second Amazon Mechanical Turk task and IRT analysis confirmed that the 55 questions test a latent ability dimension for EHR note comprehension. A short test of 14 items was created along with the 55-item test.

**Conclusions:**

We developed ComprehENotes, an instrument for assessing EHR note comprehension from existing EHR notes, gathered responses using crowdsourcing, and used IRT to analyze those responses, thus resulting in a set of questions to measure EHR note comprehension. Crowdsourced responses from Amazon Mechanical Turk can be used to estimate item parameters and select a subset of items for inclusion in the test set using IRT. The final set of questions is the first test of EHR note comprehension.

## Introduction

### Background and Significance

Providing patients access to their medical records through personal health records (PHRs) is becoming more common as physicians move to electronic health record (EHR) systems. PHRs are defined as “electronic, lifelong resource of health information needed by individuals to make health decisions” [1]. Providing patients direct access to their EHR clinical notes can enhance patients’ understanding of their clinical conditions and improve their health care outcomes [2-4]. For example, the Veterans Health Administration offers the My Health*e*Vet PHR through a Web-based patient portal, which allows millions of veterans to view their EHRs [5]. These records include both structured (eg, patient vitals) and unstructured data (eg, discharge summaries and clinical notes). However, patients with limited health literacy may struggle to understand the content of their medical notes, which can include visit summaries with medical terms, lab reports, and terms and phrases that are not common outside of medicine. A patient’s health literacy can have an impact on their desire to engage with their own PHR [6,7].

Low health literacy can impact a patient’s ability to communicate with their health care providers and to navigate and understand complex EHR information. Health literacy is defined by the Institute of Medicine as “the degree to which individuals have the capacity to obtain, process, and understand basic information and services needed to make appropriate decisions regarding their health” [8]. According to the National Assessment of Adult Literacy, only 12% of adults are proficient in health literacy [9]. The average American reads at or below an eighth grade level, and over 90 million Americans have limited health literacy [9]. Moreover, 50% of patients do not understand at least one term in their medical problem list [8,10,11]. In addition, EHR notes do not align well with existing readability prediction formulas, making it difficult to estimate EHR note readability [12]. Consider the following example, taken from a de-identified EHR clinical note: “The *monitor* has not shown any *dysrhythmias* or *arrhythmia* either *prior to or during any of his spells.* ” A patient might struggle to understand the medical terms *dysrhythmias* and *arrhythmia* and might not understand what the *monitor* is or what *prior to or during any of his spells* is referring to.

Low health literacy can lead to serious problems. For example, low health literacy was shown to be independently associated with an increase in mortality among the elderly [13]. A recent assessment of health literacy involving over 400 Veterans found that 87% of Veterans have low health literacy [14]. Most health care consumers do not understand phrases often used in cancer consultations [15]. Patients understand less than 30% of medical terms commonly used in the emergency department [16]. Patients with low health literacy are more likely to lack awareness of their atrial fibrillation diagnosis [17] and are at higher risk for increased fear of cancer progression [18].

### Objective

Given the prevalence of low health literacy in the population, tools that effectively assess a patient’s health literacy are needed for both research and practice. Of the existing instruments, 3 that are widely used are the Rapid Estimate of Adult Literacy in Medicine (REALM), the Test of Functional Health Literacy in Adults (TOFHLA), and the Newest Vital Sign (NVS) [19-21]. Each of these has value, but also limitations. For example, REALM can be administered in 2 to 3 min, but it assesses word recognition, not comprehension [19]. TOFHLA assesses reading comprehension and numeracy using passages from health care–related documents, hospital forms, and prescription labels [20]; a short version of TOFHLA reduced the administration time from 22 min to 12 min [22]. NVS contains 6 items tied to a single stimulus (a food label) and can be administered in 3 min. It was intended as a screening tool and is less appropriate for generating scores that discriminate between different levels of health literacy in patients [21,23]. Taken together, these tests can provide information on a patient’s general health literacy, but none assesses a patient’s ability to comprehend EHR notes.

The purpose of this study was to create an instrument to measure EHR note comprehension in patients. We first identified a set of representative EHR notes for 6 diseases and conditions from a large hospital EHR system. From these notes, a group of physicians and medical researchers generated questions using the Sentence Verification Technique (SVT) [24-26]. We obtained responses for these questions from the crowdsourcing platform Amazon Mechanical Turk (AMT) and analyzed the results using item response theory (IRT) [27-30] to select a subset of questions for a test of EHR note comprehension. To the best of our knowledge, the ComprehENotes question set is the first instrument to assess EHR note comprehension.

## Methods

### Overview

**Figure 1:**
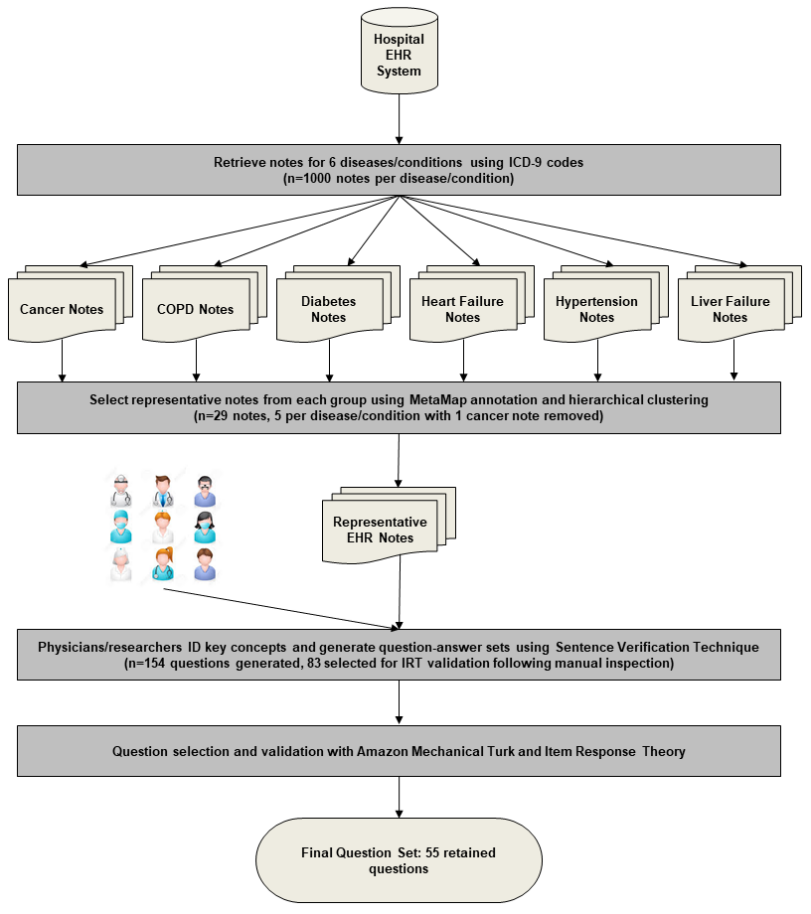
Visualization of the question generation and validation process. COPD: chronic obstructive pulmonary disease; EHR: electronic health record; ICD-9: International Classification of Disease-9; IRT: item response theory.

The goal of this work was to develop a set of questions that could be used to test patient EHR note comprehension. To that end, we developed a process for note selection, question generation, and question selection and validation (Figure 1). We discuss each step in detail in the following sections.

### Electronic Health Record Note Selection

We selected notes according to the International Classification of Disease codes associated with 6 important and common diseases: heart failure (428), hypertension (401), diabetes (249, 250), chronic obstructive pulmonary disease (COPD; 493.2, 491, 492, 494, 496, 506), liver failure (571), and cancer (140-239). By selecting notes from multiple diseases, our goal was to obtain a variety of notes associated with common diseases to generate questions across multiple topics.

We retrieved EHR discharge summary and progress notes from the University of Massachusetts Memorial Hospital EHR system. Progress notes provide information regarding a patient’s conditions and treatments, whereas discharge notes may include a summary of the patient’s visit, necessary patient follow-up, and other information. These types of notes include information that is relevant to patients and are good candidates for question generation. For each disease, we randomly selected 1000 notes. As the EHR notes vary significantly in length (anywhere from 50 words to over 1500 words), we limited the note selection to notes between 300 and 1000 words long. Notes that are longer than 1000 words often contain duplicate information or large tables of lab results, with few free-text section from which we can generate questions. We annotated each note with the MetaMap [31], a toolkit developed by the National Library of Medicine, to map the note to Unified Medical Language System (UMLS) concepts [32]. For each category, we ran topic modeling on the 1000 notes using the UMLS concepts that were identified by MetaMap and hierarchically clustered the notes into 5 clusters based on topic similarities. Finally, we selected 1 representative note (the note with the most UMLS concepts) from each cluster. By selecting the note with the most concepts, our goal was to identify those notes with the most information that could be used as part of the question generation process. This procedure resulted in a total of 30 notes, with 5 notes per disease. We discarded 1 cancer note because the physicians identified it as a pure lab test report that did not include any natural language text.

### Generating Questions With Sentence Verification Technique

We asked experts to create question-answer sets by following these 2 steps: (1) identifying important content in the notes and (2) creating comprehension test questions. Specifically, the selected 29 de-identified notes were provided to 5 groups. Each group included 1 physician and 2 to 3 nonclinician researchers (a total of 4 physicians and 13 researchers, where 1 physician participated in 2 groups). The groups were given an introduction to the SVT methodology before taking part in the exercise. Each member read every assigned EHR note and then identified important content (usually a sentence). Each member then followed the SVT protocol to create question-answer sets for the identified content.

SVT is a procedure for generating reading comprehension items to evaluate whether an individual has understood a passage of text [24,33,34]. SVT has been applied in many different reading comprehension environments, such as basic language research [35], evaluating the effect of prior beliefs on comprehension [36], and assessing language skills of non-native English speakers [37]. In addition, SVT has been used to develop tests to assess comprehension of cancer screening and prevention messages [25,26]. SVT tests are sensitive to both differences in reading skill and text difficulty. Tests using SVT questions have been shown to be effective for measuring reading comprehension and for assessing comprehension of written and spoken health messages [25,26].

An SVT test is designed by taking a sentence or phrase from a passage of text (the *original*) and generating 3 additional sentences or phrases: (1) a *paraphrase*, where as much of the sentence or phrase is changed as possible while preserving the original meaning, (2) a *meaning change*, where the original sentence or phrase is changed slightly but enough that the original meaning is changed, and (3) a *distractor*, which is unrelated to the original but still consistent with the passage theme [24].

Once generated, the question-answer sets were then discussed in the group and a final question-answer set was agreed upon. From the 29 EHR notes, 154 question-answer sets were generated. Table 1 shows examples of question-answer sets generated by the groups, and Textbox 1 shows how these questions would be presented to patients in a test scenario. We selected 83 of the 154 questions for further analysis. Questions were selected based on their content. We manually selected questions that were generally relevant to the main topic (eg, diabetes) over questions that were very specific to a patient’s note to keep the question set general enough to be given to future patients. We retained 11 to 13 question-answer sets for 4 of the 6 topics and 18 question-answer sets for COPD and diabetes.

### Data Collection

To gather enough human responses to fit the IRT model, we recruited participants from AMT. AMT is a Web-based microtask crowdsourcing platform where individuals (called Turkers) perform Human Intelligence Tasks (HITs) in exchange for payment. HITs are usually pieces of larger, more complex tasks that have been broken up into multiple, smaller subtasks. AMT and other crowdsourcing platforms are used to build large corpora of human-labeled data at low cost compared with using expert annotators [38,39]. Researchers’ projects have used AMT to complete a variety of tasks [40,41]. Recent research has shown that AMT and other crowdsourcing platforms can be used to generate corpora for clinical natural language processing and disease mention annotation [41,42]. AMT was used to detect errors in a medical ontology, and it was found that the crowd was as effective as the domain experts [43]. In addition, AMT workers were engaged in identifying disease mentions in PubMed abstracts [42] and rank adverse drug reactions in order of severity [44] with good results.

We created 6 comprehension tasks on AMT, 1 per disease topic, to analyze each topic separately. Each task was completed by 250 Turkers, who were presented with the test questions, 1 question at a time. This sample size is large enough to satisfy the accepted standards for IRT models based on the noncentral chi-square distribution [45]. We collected demographic information from the Turkers before administering the test questions, and we implemented several quality control mechanisms to ensure the quality of the Turker results. Only Turkers with approval rates above 95% and located in the United States were able to participate. The 95% approval rate identifies Turkers who have been approved most of the time according to their completion of other tasks on AMT and is indicative of the high quality of their previous tasks. Restricting the task to users located in the United States is used as a proxy for English proficiency. In addition, in each test, 1 question was randomly selected as a quality-check question and was presented to the Turker twice during the course of the evaluation. If the Turker gave 2 different answers to the repeated question, their responses were not included in later analyses. Two simple questions were also added to the test as quality control. If the Turker answered 1 or both of the quality control questions incorrectly, their responses were rejected from consideration and not included in later analyses.

For the COPD and diabetes tests, the 18 questions were split into 3 groups of 6 questions. Each Turker was given a random selection of 2 of the 3 groups. In this way, the test lengths were similar to the other disease topic tests, and the conditions in which Turkers provided responses were consistent across the groups. For the COPD and diabetes tasks, we recruited 400 Turkers so that the number of responses per question was consistent with the other topics.

**Table 1:** Examples of questions generated from the researcher and physician groups.

Original statement from EHR^a^ notes	Paraphrase	Meaning change	Distractor
The monitor has not shown any dysrhythmias or arrhythmia either before or during any of his spells	His heart rhythm is normal before and during his fainting spells	He has had abnormal rhythm before or during his spells of chest pain	The monitor has shown abnormal heart rhythms before and during his spells
Patient recently presented to the hospital with shortness of breath	She went to the hospital for trouble breathing	She visited the clinic due to shortness of iron	Shortness of breath has many causes

^a^EHR: electronic health record.

**Examples of how the generated questions would be displayed as a questionnaire, using the examples from Table 1.**

Please read the following question and then examine the answer choices and choose the answer that best represents the question text.

What does the following sentence mean? “The monitor has not shown any dysrhythmias or arrhythmia either prior to or during any of his spells.”

1. He has had abnormal rhythm before or during his spells of chest pain.
2. The monitor has shown abnormal heart rhythms before and during his spells.
3. His heart rhythm is normal before and during his fainting spells.

What does the following sentence mean? “Patient recently presented to the hospital with shortness of breath.”

1. Shortness of breath has many causes.
2. She went to the hospital for trouble breathing.
3. She visited the clinic due to shortness of iron.

### Item Analysis and Selection Using Item Response Theory

After data collection, the Turker responses were analyzed using a 3-parameter logistic (3PL) IRT model. IRT [27,46] is one of the most widely used approaches for item evaluation and test construction [29,30,47]. For example, the Patient Reported Outcomes Measurement Information System funded by the National Institutes of Health has used IRT to characterize item banks and to support computerized adaptive testing [28].

In IRT, a statistical model jointly models an individual’s responses to individual test items with a person’s ability level and the item’s features [27]. IRT models make several assumptions: (1) people differ from each other on an unobserved latent dimension of interest (usually called *ability*); (2) the probability of correctly answering a particular item is a function of the latent ability dimension (the item characteristic curve, ICC); (3) responses to individual items are independent of each other for a given ability level of a person (the *local independence assumption*); and (4) responses from different individuals are independent of each other. There are a variety of IRT models; one of the models widely used is the 3PL model. In the 3PL model, ICC is assumed to follow a logistic function with a nonzero lower asymptote:

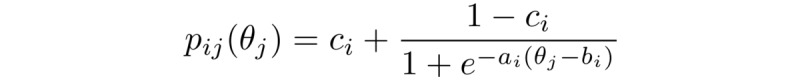



In the above equation, *p*_ij_ is the probability that person *j* answers item *i* correctly, and *θ*_j_ is the ability level of individual *j*. In this work, *θ* represents the ability of an individual on the task of EHR note comprehension. As individual persons are assumed to be sampled from a population, their ability levels are assumed to be a random effect with a normal distribution. There are also 3 item parameters: the guessing parameter *c*_i_ is the lower asymptote of the ICC curve and represents the probability of guessing, the difficulty parameter *b*_i_ is the level of ability that produces a chance of correct response equal to the average of the upper and lower asymptotes, and the slope or discrimination parameter *a*_i_ is related to the steepness of the curve.

The 3PL model was fit to data for each set of questions using the open source software R packages *mirt* and *ltm* [48,49]. Marginal residuals of each pair of items and each triplet of items were checked, and items that gave large residuals were removed for violation of local independence. Items with a negative slope were also removed. Guessing parameters not significantly different from 0 were set to 0. A key parameter used to identify a good question for future evaluations is the slope of ICC. If the slope is flat, then the item cannot distinguish between individuals of high ability levels and individuals of low ability levels. After refitting the remaining items, items with a slope parameter not significantly greater than 0 or less than 0.71 were removed. The value 0.71 corresponds to a communality of 0.15 in an exploratory factor analysis, which means that 15% of the variance of the item would be explained by the latent ability factor if the item were continuous. We retained 55 items in this analysis for further validation. From the 55 items, we also identified 14 of the 55 items with the largest slopes (discrimination parameters) and highest average information for inclusion in the short form of the test. The short test should be as informative as possible while reducing the length of the test, making it more practical to administer.

### Confirmatory Evaluation of Item Quality Using Item Response Theory

The questions retained from the initial IRT analysis were combined into a single test and deployed in a new AMT task to validate the item parameters. For this task, we split the 55 retained questions into 3 groups (each of 18-19 questions) and created 3 AMT tasks in which Turkers were shown 2 of the 3 groups and asked for responses as above. Quality checks were included as in the first set of AMT tasks. For these tasks, Turkers who participated in the initial data collection were excluded. Responses were generated and a second round of IRT analysis was performed to confirm that the questions retained from the first round could be considered a cohesive test of EHR note comprehension as a whole.

## Results

### Amazon Mechanical Turk Responses and Turker Demographics

**Figure 2:**
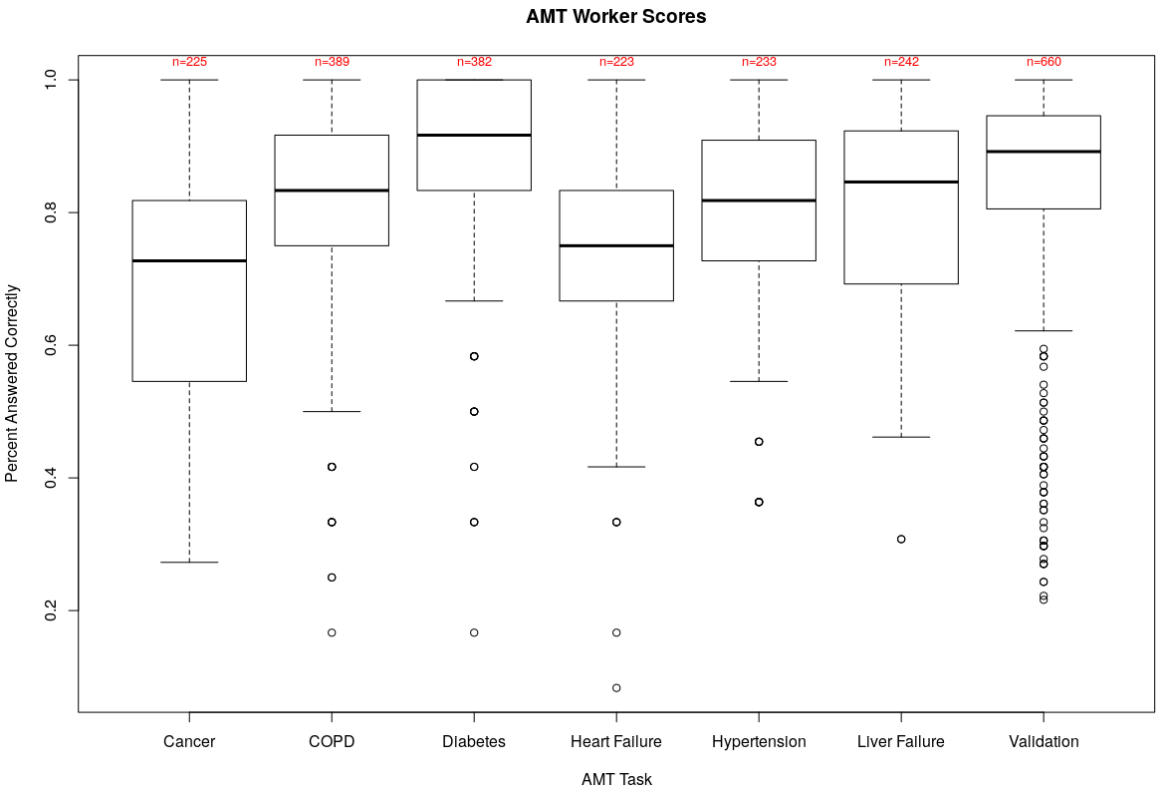
Box plots of Turker scores on the AMT per-topic and validation tasks. The center rectangles span the range from the first quartile to the third quartile of responses, and the bolded line inside each box represents the median score. Open circles indicate outlier scores. In the cancer plot, the upper and lower horizontal lines indicate the maximum and minimum scores, respectively. For all others, the lower horizontal line is 1.5 times the interquartile range below the first quartile. Average raw score is above 69% in all cases. Counts indicate the number of AMT responses retained after quality-control. AMT: Amazon Mechanical Turk; COPD: chronic obstructive pulmonary disease.

We first report descriptive statistics and demographic information about the Turkers who completed the per-topic and validation AMT tasks (Figure 2; Table 2). Responses for both the per-topic and validation tasks covered a wide range of correctly answered questions. Mean scores for the cancer, COPD, diabetes, heart failure, hypertension, liver failure, and validation tasks were 69% (7.6/11), 78% (9.4/12), 88% (10.6/12), 70% (8.4/12), 78% (8.6/12), 79% (10.3/13), and 85% (31.4/37), respectively. Across all tasks, no more than 10.8% (27/250 for the heart failure task) of responses were removed because of quality control checks.

We also looked at raw scores and estimated ability in the validation task to see whether there were patterns in the responses that matched expected behavior (Table 3). As expected, mean scores for individuals with more education are higher than for individuals with less education. In addition, Turkers over 45 years score higher on average than Turkers under 45 years. There is a slight drop in mean scores for Turkers aged over 65 years, which makes sense given that adults aged 65 years and older have lower health literacy on average [9].

### Item Response Theory Analysis

#### Item Selection Using Item Response Theory

**Figure 3:**
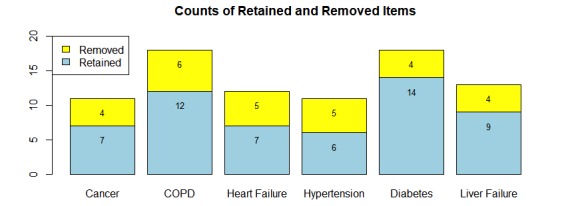
Results of analysis to identify useful items from the question sets. Items were removed according to the reasons outlined in the Methodology section. COPD: chronic obstructive pulmonary disease.

Of the 83 questions provided to Turkers in the per-topic AMT tasks, 55 (66%) were retained after the initial IRT analysis (Figure 3). Items were identified for removal according to the procedure identified in the Methods section.

**Table 2:** Demographic information of Turkers from the per-topic and validation Amazon Mechanical Turk tasks.

Demographic characteristic		Per-topic tasks count (N=1694), n (%)	Validation task count (N=660), n (%)
**Gender**			
	Male	880 (51.95)	250 (37.9)
	Female	814 (48.05)	411 (62.1)
**Race**			
	African American	107 (6.32)	58 (8.8)
	Asian	163 (9.62)	51 (7.7)
	Hispanic	89 (5.25)	32 (4.8)
	American Indian	7 (0.41)	12 (1.8)
	Pacific Islander	9 (0.53)	0 (0)
	White	1319 (77.86)	507 (76.8)
**Highest level of education**			
	Less than high school	17 (1.00)	4 (0.6)
	High school degree	504 (29.75)	189 (28.6)
	Associate’s degree	283 (16.71)	108 (16.4)
	Bachelor’s degree	697 (41.15)	256 (38.8)
	Master’s degree or higher	193 (11.39)	103 (15.6)
**Age in years^a^**			
	18-21	N/A^b^	12 (1.8)
	22-34	N/A	330 (50.0)
	35-44	N/A	158 (23.9)
	45-54	N/A	106 (16.1)
	55-64	N/A	39 (5.9)
	65 and older	N/A	15 (2.3)

^a^Age demographic information was not collected as part of the per-topic Amazon Mechanical Turk tasks.

^b^N/A: not applicable.

Table 4 shows examples of retained and removed items. In the case of the removed item, the question simply defining the term *Osteoporosis* was too easy for the Turker population. That is, most of the Turkers answered the question correctly, and thus, the probability of answering the question correctly is very high even at low levels of ability. A question like this does not give us any information about an individual’s ability and therefore is not needed in the test set.

**Figure 4:**
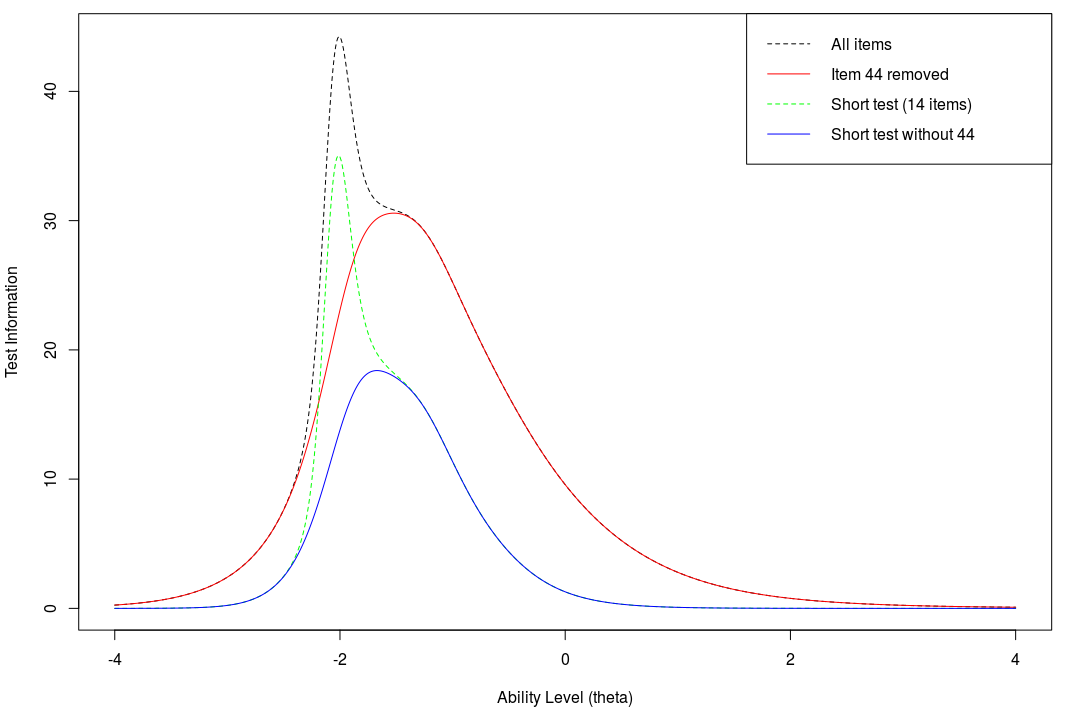
Test information curve for the full ComprehENotes instrument (55 items) and various subsets.

The test information curve is presented in Figure 4. Test information is defined as the reciprocal of the squared SE of the ability estimate: *I*=1/ *σ*^2^, where σ is the SE [27]. Test information measures how accurate the ability estimates are at varying levels of ability. Given that most items have negative difficulty, the information curve has high values in the negative ability levels. That is, estimates of ability for negative ability levels are more accurate. Test information is greater than 4 for the range of ability levels between −2.8 and 0.7, which means for this range of ability levels (from 2.8 SDs below to 0.7 SD above the average of the population of AMT users), SE of an ability estimate is smaller than 0.5. The full test is most informative in ability around −2 with maximum information of 44.2 (Figure 4, red dotted line). This maximum is mostly because of a single item (44) with the largest slope of 11.3. Due to the very large slope parameter, this item is very informative around ability of −2 but is not informative at other areas of ability. As one goal of the test is to identify individuals with low ability, this item may be useful and is therefore included in our test set. However, we also wanted to confirm that the other test questions are still informative in their own right. To do this, we plotted the test information curve without item 44. Without this item, the item information curve is most informative around −1.5, with a maximum of 30.6 (Figure 4, black solid line). The maximum information of each item, its location in the ability spectrum, and the average information in the range between −4 and 4 are also summarized in Multimedia Appendix 1. The test information curve of the short test is also presented in Figure 4. The short test includes item 44, and thus, we also plot information for a 13-item test without item 44. For the short test, test information is greater than 4 (ie, SE of ability estimate is smaller than 0.5) in the range between −2.4 and −0.5, or 2.4 SDs to 0.5 SD below the average AMT user, again appropriate for a population of low literacy.

**Table 3:** Average estimated ability of Turkers according to demographic information for the validation task.

Demographic characteristic		Mean correct, %	Average estimated ability
**Education**			
	Less than high school	64.7	−0.899	
	High school degree	84.9	−0.038
	Associate’s degree	83.8	−0.013
	Bachelor’s degree	83.8	−0.034
	Master’s degree or higher	88.1	0.199
**Age in years**			
	18-21	77.4	−0.493
	22-34	83.7	−0.042
	35-44	83.6	−0.066
	45-54 88.3	0.222	
	55-64	89.4	0.212
	65 and older	85.9	−0.122
**Gender**			
	Male	80.6	−0.236
	Female	87.2	0.143

**Table 4:** Examples of retained and removed questions following item response theory analysis.

Item Retention Decision	Question	Paraphrase	Meaning change	Distractor
Retained	Pegfilgrastim 6 mg subcutaneous one dose	Do an under skin injection of one dose of 6 mg pegfilgrastim	Pegfilgrastim 6 mg epidermal one dose	Pegfilgrastim may prevent neutropenia
Removed	Osteoporosis	Weakness in bones	Hardening of bones as we get older	Some bones get hard and some weak

## Discussion

### Principal Findings

The goal of this project was to develop an instrument to assess patients’ ability to comprehend content in EHR notes. To that end, we developed a process for identifying relevant EHR notes, creating a large question set and reducing the question set to a reasonable size using IRT. We generated questions from EHR notes using SVT and administered them to a population of crowd workers using AMT. We then used IRT to estimate the item parameters and select a subset of items for our instrument. The final test measures a patient’s ability to read and comprehend EHR notes. These questions are general enough to be applicable to a wide variety of individuals while still being grounded in specific medical concepts as a result of the hierarchical clustering process.

In contrast with existing tests of health literacy, ComprehENotes was developed by generating questions directly from real patient de-identified EHR notes. Key concepts from the notes were identified by physicians and medical researchers as part of the question generation process. These concepts were deemed important for patients to understand, and the test questions were designed to assess comprehension of these concepts. The ComprehENotes test is the first to directly assess a key element of health literacy, that is, the ability to read and comprehend EHR notes. (To obtain the test, please contact the authors.)

The test is most informative at low levels of ability (Figure 4), which is consistent with our long-term goal of identifying patients with low EHR note comprehension ability. Although the test was easy for the AMT workers, the demographics show that those individuals are not representative of demographics at higher risk of low health literacy (eg, low education and the elderly). Those AMT workers who did fit in the demographics that are more likely to have low health literacy did perform worse in terms of average ability (Table 3). The number of Turkers in those groups was low compared with other demographic groups (Table 2), and thus, more evaluation with individuals with higher likelihood of low health literacy is required. Most of the questions have low difficulty estimates, which makes the test appropriate for screening for low health literacy. It is important to note that the ability estimates are based on the responses of the AMT workers. If we were to fit a new IRT model using response patterns from a patient population, ability estimates of future test takers would be with respect to the patient population. This does not affect the test itself but only how the ability estimates are interpreted. Using the test as developed here, new response patterns are scored and compared with the average AMT user.

We also identified items from our instrument that can be used in a short test to reduce administration time while still being informative. The short test reduces the number of items from 55 to 14 while still being very informative at low levels of ability. This short test can be administered more quickly than the full test while still being informative at low levels of ability.

### Limitations

There are limitations with this work. Fitting IRT models requires a large number of human responses to a relatively small number of questions. The length of the question set must be short to avoid a drop in response quality due to boredom or fatigue. Although the cost of gathering a large number of responses is reduced by using AMT or other crowdsourcing platforms, scaling the number of questions that can be analyzed with IRT remains a challenge.

The groups of physicians and medical researchers who generated our question sets are not experts in question generation using SVT. However, before the task, they all received training on what SVT is and how to construct questions using the methodology. In addition, we manually selected a subset of the questions that were generated for IRT analysis and validation. In this way, we were able to identify a set of questions that could be generalized to a test set. The IRT validation confirmed that a set of questions was appropriate as a test of EHR note comprehension.

The demographics of Turkers that took part in our tasks are not representative of the entire US population, and in particular, do not cover groups with low average health literacy (eg, minorities, people with less than a high school degree, older adults) [9]. However, all but 1 of the questions included in the final question set have difficulty parameters less than or equal to 0. These questions therefore will be appropriate to test ability for individuals with low EHR note comprehension ability. Future work should validate that the questions are in fact appropriate for individuals with low health literacy.

The full ComprehENotes test is long at 55 questions. The length makes it impractical to administer in clinical settings because of the time needed to complete the test. However, we have also identified a short test of 14 items that can be administered in a short period of time. The 14-item test includes items with the largest slope parameters and average information. The short test is still informative at levels of ability below 0, which is appropriate given that the goal of developing this test was to identify individuals with poor EHR note comprehension ability.

### Conclusions and Future Work

The ComprehENotes question set is an instrument for measuring EHR note comprehension. Validation of the metric as compared with existing tests of health literacy is still required. During a pilot version of our AMT task, we asked participants to complete the Short Test of Functional Health Literacy in Adults (STOFHLA) as well as our test and found that all the respondents scored a perfect score (36) or answered 1 question wrong on the STOFHLA and were therefore considered to have Adequate Health Literacy according to the STOFHLA scoring. Comparing this metric to existing tests such as REALM or TOFHLA in a population with low health literacy is an important future work to validate the metric as a valid measure of health literacy. In addition, further analysis of how different groups perform on this question set can inform how EHR notes are provided to patients and what types of educational materials should be provided to patients.

The ComprehENotes test can be administered to patients as is to assess EHR note comprehension ability. As the questions are associated with certain diseases and conditions, subsets of the test can also be administered independently to test EHR note comprehension in specific patient populations. For example, the questions associated with liver failure can be extracted and administered as a standalone test to assess EHR note comprehension in liver failure patients. In this way, questions specific to certain diseases can be used to test comprehension among patient populations where the terms are more likely to appear.

Finally, this work is a first step toward being able to evaluate patients’ understanding of their health based on information directly contained in their own EHR. We have shown that it is possible to develop a test of health literacy from questions obtained from EHR notes. Automating steps of the question generation and validation processes with clinical natural language processing tools are interesting directions for future work. For example, one such step would be to build an NLP model to generate questions for a specific patient given his or her own EHR note text. The model can be trained on the ComprehENotes questions to identify information that would be relevant for generating good questions. These personalized questions can be administered to patients to evaluate their ability to read and comprehend their own notes.

## Appendix

Multimedia Appendix 1 Table of item parameter estimates and item information in the validation sample.

## Abbreviations

AMT: Amazon Mechanical Turk
COPD: chronic obstructive pulmonary disease
EHR: electronic health record
HIT: Human Intelligence Task
ICC: item characteristic curve
IRT: item response theory
NVS: Newest Vital Sign
PHR: personal health record
REALM: Rapid Estimate of Adult Literacy in Medicine
SVT: Sentence Verification Technique
TOFHLA: Test of Functional Health Literacy in Adults
UMLS: Unified Medical Language System
3PL: 3-parameter logistic
