# Supplementary material for: ComprehENotes, an Instrument to Assess Patient Reading Comprehension of Electronic Health Record Notes: Development and Validation
Source: J Med Internet Res. 2018 Apr 25;20(4):e139. doi: 10.2196/jmir.9380 (PMC5943623; doi:10.2196/jmir.9380)
Supplement: Multimedia Appendix 1 [file jmir_v20i4e139_app1.pdf]

Multimedia Appendix 1: Item parameter estimates and item information in the validation sample.

Item**	Slope*	Difficulty	Communality	Max. Information	Max. Location	Average Information
1	1.0	-1.1	0.26	0.18	-0.79	0.08
2^	3.1	-0.8	0.77	1.70	-0.72	0.24
3	1.3	-0.9	0.36	0.27	-0.65	0.09
4	1.4	-1.6	0.40	0.33	-1.43	0.1
5^	5.0	-1.9	0.90	4.36	-1.84	0.38
6^	2.6	-1.1	0.70	1.15	-1.03	0.19
7	1.5	-0.3	0.42	0.36	-0.11	0.11
8	2.3	-0.5	0.64	0.87	-0.41	0.17
9	1.5	-1.0	0.45	0.40	-0.78	0.11
10	1.6	-2.1	0.46	0.42	-1.91	0.12
11	1.4	-0.6	0.40	0.33	-0.43	0.1
12	1.9	-2.0	0.56	0.64	-1.89	0.14
13^	2.4	-1.4	0.67	0.99	-1.30	0.18
14***	0.6	-1.9	0.12	0.07	-1.45	0.04
15	1.3	-0.9	0.38	0.31	-0.67	0.1
16^	3.1	-1.9	0.76	1.59	-1.84	0.23
17	1.3	-1.9	0.36	0.28	-1.73	0.09
18	1.6	-1.2	0.48	0.46	-1.01	0.12
19	1.2	-1.5	0.32	0.23	-1.23	0.09
20	1.3	-2.2	0.38	0.31	-2.05	0.1
21^	2.7	-1.7	0.72	1.29	-1.58	0.21
22	1.7	-2.0	0.49	0.47	-1.80	0.12
23^	2.6	-1.8	0.70	1.17	-1.66	0.2
24	1.7	-1.3	0.49	0.47	-1.17	0.12
25	1.8	-2.1	0.54	0.58	-1.94	0.14
26	1.1	-0.4	0.31	0.22	-0.13	0.08
27	1.4	-1.6	0.41	0.35	-1.43	0.11
28	1.8	-1.2	0.53	0.56	-1.07	0.14
29^	3.6	-1.8	0.82	2.22	-1.72	0.27
30***	0.8	-0.7	0.19	0.12	-0.37	0.06
31^	2.4	-1.8	0.66	0.97	-1.66	0.18
32	1.7	-1.7	0.49	0.48	-1.56	0.13
33	1.7	0	0.49	0.47	0.12	0.12
34^	3.1	-2.0	0.77	1.69	-1.94	0.24
35	1.3	-0.7	0.36	0.28	-0.47	0.1
36	2.0	-1.3	0.59	0.70	-1.14	0.15
37	1.5	-1.6	0.45	0.40	-1.40	0.12
38	1.4	-2.1	0.40	0.32	-1.94	0.1

Item**	Slope*	Difficulty	Communality	Max. Information	Max. Location	Average Information
39	2.0	-1.0	0.58	0.70	-0.88	0.15
40	2.2	-0.8	0.63	0.83	-0.73	0.17
41***	0.5	0.7	0.07	0.04	1.21	0.03
42	1.3	-0.8	0.36	0.28	-0.60	0.10
43	2.1	-1.5	0.59	0.72	-1.32	0.15
44***^	11.3	-2.1	0.98	21.87	-2.03	0.85
45	1.8	-2.0	0.52	0.55	-1.88	0.13
46	1.8	-1.5	0.53	0.55	-1.35	0.13
47^	4.3	-1.3	0.87	3.20	-1.20	0.32
48	1.7	-1.9	0.51	0.52	-1.72	0.13
49	1.6	-1.2	0.47	0.44	-1.01	0.12
50^	4.2	-1.4	0.86	2.97	-1.34	0.31
51	2.1	-0.8	0.60	0.75	-0.65	0.16
52^	3.9	-1.8	0.84	2.61	-1.77	0.29
53	1.4	-0.7	0.39	0.31	-0.49	0.10
54	1.5	-0.6	0.45	0.41	-0.40	0.12
55	1.9	-1.8	0.56	0.62	-1.64	0.14
Test				44.24	-2.01	8.77
Test w/o 44				30.58	-1.52	7.92
Selected items				35.04	-2.01	4.08
Selected w/o 44				18.40	-1.67	3.23

*All slope parameters are significant after Holm's adjustment of multiplicity.

**The constant guessing parameter has an estimate of 0.20, significantly different from zero.

*** These four items have extreme slope or intercept estimates.

^ Selected 14 items for short test.
